# Accumulation of AGO2 Facilitates Tumorigenesis of Human Hepatocellular Carcinoma

**DOI:** 10.1155/2020/1631843

**Published:** 2020-04-30

**Authors:** Yang Yang, Qi Mei

**Affiliations:** ^1^Department of Ophthalmology, Renmin Hospital of Wuhan University, No. 9 ZhangZhiDong Street, Wuhan, China; ^2^Department of Oncology, Tongji Hospital, Tongji Medical College, Huazhong University of Science and Technology, No. 1095 Jie Fang Avenue, Wuhan, China

## Abstract

AGO2 (Argonaute RISC Catalytic Component 2) plays an important role in small RNA-guided gene silencing processes. It has been implied in tumorigenesis of different types of tumors. In this study, we found that AGO2 expression was remarkably increased in human hepatocellular carcinoma (HCC) tissues when compared with adjacent noncancerous tissues. High expression of AGO2 was associated with poor prognosis in HCC patients. The CRISPR/Cas9-mediated knockout of AGO2 in SMMC-7721 cells inhibited cell proliferation and induced significant G1 phase arrest of cell cycle. Inhibition of cell migration was also observed in SMMC-7721 *AGO2*^−/−^ cells. *In vivo* experiments showed that tumors grew slower in nude mice transplanted with *AGO2*^−/−^ cells than in SMMC-7721 cell-derived xenograft mice. Microarray analysis and western blot analysis revealed that AGO2 depletion decreased expression of Survivin, Vimentin, and Snail. Overexpression of AGO2 in SMMC-7721 and Huh-7 cells could reverse the knockout-induced inhibition effects on either cell behaviors or expression of Survivin, Vimentin, and Snail Therefore, our data demonstrated that AGO2 might facilitate HCC tumorigenesis and metastasis through modulating expression of Survivin, Vimentin, and Snail.

## 1. Introduction

Hepatocellular carcinoma (HCC), a malignant epithelial liver tumor, is the third leading cause of cancer-associated death globally [1]. The development of HCC is a multistage process involving chronic liver injury, inflammation, hepatocellular degeneration/regeneration, necrosis, and small-cell dysplasia [2]. Genetic and epigenetic aberrations are implied in HCC initiation and progression [2].

Argonaute (AGO) proteins are expressed at high levels in a wide range of organisms. The human Argonaute family has eight members, four of which belong to the eIF2C/AGO subfamily. These eIF2C/AGO subfamily members are essential components of the RNA-induced silencing complex (RISC), which is involved in RNA silencing processes of RNA interference (RNAi) [3]. Among them, only AGO2 has intrinsic endonuclease activity. AGO2 can bind small interfering RNA (siRNA) or microRNA (miRNA), which guides AGO2 to target specific messenger RNA (mRNA) and cause the mRNA cleavage [4, 5]. It has been well documented that AGO2 plays important roles in multiple biological or pathological processes [6–8]. Recently, AGO2 has been demonstrated as a potential oncogene in human tumorigenesis, including head and neck squamous cell carcinoma [9], nasopharyngeal carcinoma [10], bladder cancer [11], and glioma [12]. It was also reported that AGO2 could promote tumor metastasis [13] and enhance angiogenesis of HCC [14]. However, the underlined mechanisms of AGO2 in HCC progression still need to be clarified.

In the present study, we found that high expression of AGO2 was an independent prognostic indicator for HCC patients with poor outcome. *In vitro* and *in vivo* studies would further reveal the function and molecular mechanisms of AGO2 in HCC tumorigenesis and progression.

## 2. Materials and Methods

### 2.1. Patients

On institutional review board approval, we identified 90 patients with hepatocellular carcinoma (HCC) treated with surgery between 2011 and 2019 at Renmin Hospital of Wuhan University and Tongji Hospital of Huazhong University of Science and Technology. None of the patients received adjuvant therapy. Data collected from each patient included gender, age at diagnosis, grade, stage, and overall survival time. Pairs of cancer tissues and adjacent epithelium tissues from the same HCC patients were obtained by surgical removal. The study was approved by the Ethics Committee of Renmin Hospital of Wuhan University (approval No.: WDRY2018-K024). Informed consent (written or verbal) was obtained from the patients in this study. All the samples were anonymous.

### 2.2. Antibodies

Primary antibodies against AGO2 (ab186733) and Survivin (ab469) were purchased from Abcam Inc. (Cambridge, UK). Antibodies for detecting Snail (#3895) and Vimentin (#5741) were purchased from Cell Signaling Technology, Inc. (Danvers, MA, USA). Primary antibody for GAPDH (sc-25778) and secondary antibodies including anti-rabbit IgG (sc-2004) and anti-mouse IgG (sc-2005) were purchased from Santa Cruz Biotechnology, Inc. (Dallas, TX, USA).

### 2.3. Tissue Microarray (TMA)

The TMA slide HLiv-HCC180Sur-04 (Outdo Biotech Co., Ltd., Shanghai, China) contained 90 cases of HCC tissues and paired para-carcinoma tissues. The formalin-fixed and paraffin-embedded tissue slides were stained by hematoxylin and eosin according to standard protocols. The target tissue cores were then labeled and punched (Beecher Instruments Inc., Silver Spring, MD, USA) with a diameter of 1.5 mm and a thickness of 4 *μ*m. A total of 180 cores were arrayed on the recipient block.

### 2.4. Immunohistochemistry

The HCC sections were washed with xylene and then rehydrated in a series of ethanol (100%, 90%, 80%, and 70%) before being fully hydrated in deionized water. After antigen retrieval using citrate buffer, the tissue sections were immersed in 0.3% hydrogen peroxide solution to inactivate endogenous peroxidase and subsequently incubate with 5% BSA for blocking non-specific binding. Next, the sections were incubated with the primary anti-AGO2 antibody (1 : 500) at 4°C overnight followed by incubation with an HRP-conjugated secondary antibody. After washing with PBS, the slides were visualized by staining with 3,3h-diaminobenzidine and hematoxylin. The immunoreactive score was calculated according to the staining intensity in three randomly selected fields (40x). The scores for staining intensity were as follows: 0 was negative, 1 was weak, 2 was moderate, and 3 was strong. All slides were examined and scored by two senior pathologists independently. According to the average scores (AS), high expression level of AGO2 was defined when AS > 0.5. Low expression level of AGO2 was defined when the AS ≤ 0.5.

### 2.5. Cell Lines

Several cell lines were used in this study, including human liver cancer cell lines (SMMC-7721, HepG2, Huh-7, and Hep3B), HEK293T, and human breast cancer cell lines (HCC-1937, ZR-75-30, MCF-7, and MDA-MB-231). Cells were incubated with DMEM medium (Thermo Fisher Scientific, Waltham, MA, USA) supplemented with 10% fetal bovine serum (FBS; ScienCell, Carlsbad, CA, USA). Cells were maintained in a cell incubator with 5% CO_2_ at 37°C. All cell lines were purchased from ATCC. Microsart® *Mycoplasma* Kit (Sartorius Inc., Gottingen, Germany) was used to monitor cells for *Mycoplasma* contamination routinely.

### 2.6. Construction of AGO2 Knockout Cell Line

The AGO2 in SMMC-7721 cells were knocked out by using CRISPR/Cas9- (clustered regularly interspaced short palindromic repeats-) associated nuclease Cas9 gene editing method. Single guide RNA (sgRNA) was designed to target genomic *AGO2* exon using online tools, such as CHOPCHOP (http://chopchop.cbu.uib.no/). The sequences of sgRNAs were as follows: (1) 5′-TAACGCCTGCAAGCTCACGC-3′, (2) 5′-GCGTTACACGATGCACTTTC-3′, and (3) 5′-GCCACCATGTACTCGGGAGC-3′. sgRNAs were synthesized (TSINGKE Inc., Beijing, China) and cloned into the plasmid lenti-CRISPR-v2 (Addgene plasmid # 52961), respectively, as described previously [15]. The empty vector was used as a negative control. The construct was transfected into HEK293T cells with psPAX2 and psMD.2 using Lipofectamine 2000 (Thermo Fisher Scientific). At 72 hours post transfection, the lentivirus was harvested and infected SMMC-7721 cells. After 48-hour infection, stable cell lines were generated by selection of 2 *μ*g/ml puromycin for one week. The monoclonal cell was selected and planted in a 96-well plate. Cells were then harvested and subject to genome DNA sequencing and western blot analysis to confirm the depletion of AGO2 expression.

### 2.7. Cell Transfection

The pCMV3 vector carrying human *AGO2* cDNA was obtained from Sino Biological Inc. (Beijing, China). According to the manufacturer's protocol, pCMV3-AGO2 and control pCMV3 plasmids were transfected into Huh-7 cells using Lipofectamine 3000 reagent (Thermo Fisher Scientific), respectively. After 24-48 h transfection, the expression of AGO2 protein was examined.

### 2.8. CCK8 Assay

For the detection of cell proliferation, 2000 cells were planted at each well of the 96-well plate. Fresh DMEM medium (100 *μ*l) and CCK8 solution (10 *μ*l) were added into each well of the 96-well plate following incubation at 37°C for 2 hours. The absorbance was tested at 450 nm each day.

### 2.9. Colony Formation Assay

In the colony formation assay, SMMC-7721 cells were planted in the 6-well plates at a density of 600 cells per well. The colonies were then fixed by methanol and stained by crystal violet (Sigma-Aldrich, St. Louis, MO, USA) until the colonies were visible. The number of cells formed into a clone was calculated.

### 2.10. Cell Migration Assay

In the cell migration assay, 1 × 10^5^ suspended SMMC-7721 cells in serum-free medium (600 *μ*l) placed in the upper compartment of a trans-well chamber with 8 *μ*m pores (Corning Inc., Acton, MA) in a 24-well plate. Additional 600 *μ*l medium containing 10% FBS was added to the bottom well. After 12-hour incubation, cells in the bottom wells were fixed by methanol and stained by crystal violet. The number of migration cells into bottom transwells was counted.

### 2.11. Mice and Xenograft Tumor Model

BALB/c nude mice (4-6 weeks old female) were purchased from Beijing HFK Bioscience Co., Ltd. Laboratory Animal Center. The mice were housed under specific pathogen-free (SPF) conditions in separated ventilated cages. Mice were housed with 5 mice per cage and in a climate-controlled room (25°C, 55% humidity, and 12 h light/darkness cycle). All procedures involving mice and experimental protocols were approved by the ethics committee of Tongji Hospital of Tongji Medical College and in compliance with the NIH guidelines.

Mice were randomly assigned as the control group and model group (*n* = 10 mice per group). In the model group, SMMC-7721 cells (control and AGO2 knockout) were subcutaneously injected in the flank of BALB/c nude mice with 2 × 10^6^ cells. Mice in the control group only received sterile PBS injection. About 7 days post injection, the tumor size was measured every other day. The tumor size was calculated as (length × width^2^)/2. At the indicated time, all mice were sacrificed with CO_2_ inhalation and the tumors were photographed.

### 2.12. Reverse Transcription Reaction and Quantitative Real-Time PCR

Total RNA was isolated using Trizol reagent (Thermo Fisher Scientific). cDNA was prepared by the M-MLV reverse transcriptase (Promega) and was amplified on the Applied Biosystems 7500 (Thermo Fisher Scientific, CA, USA). The primers used in the study are as follows: *AGO2* forward—5′-CCTGTATGAGAACCCAATGTC, reverse—5′-CAGCTAGTTTGAGCCCATCA; GAPDH forward—5′-AAGGCTGTGGGCAAGG, reverse—5′-TGGAGGAGTGGGTGTCG.

### 2.13. Western Blot

Cells were collected and lysed in radioimmunoprecipitation assay buffer (RIPA Buffer) containing protease inhibitors. The protein concentration was determined with the BCA kit (Thermo Fisher Scientific). The same amount (30 *μ*g) of proteins was loaded on 10% SDS/PAGE gels and transferred to a piece of the nitrocellulose membrane (0.45 *μ*M) (Amersham, Piscataway, NY). After blocking by 5% nonfat milk in TBST containing 0.05% (*V*/*V*) Tween-20 at room temperature for 1 hour, the membranes were incubated overnight at 4°C with the appropriate primary antibody (1 : 2000). Blots were then incubated at room temperature for 1 hour with a horseradish peroxidase- (HRP-) conjugated secondary antibody (1 : 20000). The peroxidase activity was detected with a chemiluminescent HRP substrate (Millipore, Billerica, MA) and imaged by a chemiluminescence system (Fujifilm LAS-4000, Tokyo, Japan).

### 2.14. Statistical Analysis

The correlation between AGO2 expression and HCC clinical parameters was calculated by Spearman's correlation analysis. Survival curves were plotted using the method of Kaplan-Meier, and the significance of observed differences was calculated with a log-rank test. Student's *t*-test was used for two paired groups. One-way ANOVA followed by post hoc Turkey's test was performed for multiple comparisons. All assays were repeated for at least three times. Data are shown as average values (mean) ± SD (standard deviation) from one representative experiment. The *P* value < 0.05 was considered statistically significant.

## 3. Results

### 3.1. AGO2 Associated with Poor Prognosis in HCC Patients

**Figure 1:**
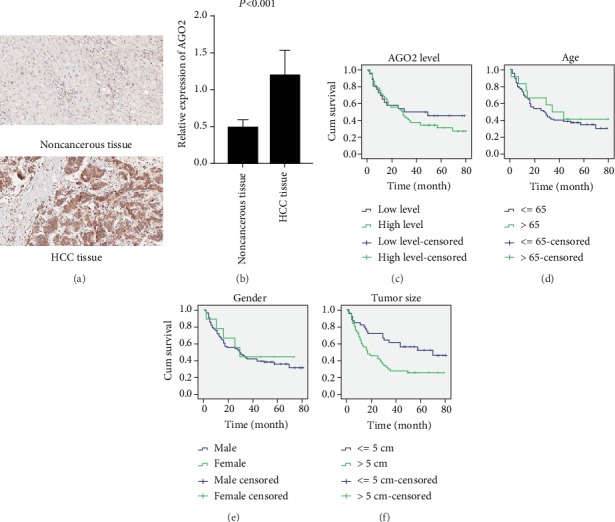
Clinical analysis of AGO2 expression in hepatocellular carcinoma patients. (a) Representative image of AGO2 IHC staining in noncancerous (epithelium) tissues and HCC tissues. (b) Relative expression levels of AGO2 in noncancerous and HCC tissues were determined by measuring the staining intensity (ratings: 0, 1, 2, and 3). Values represented as the means ± S.D. (*n* = 87). Kaplan-Meier survival analysis according to AGO2 expression (c), age (d), gender (e), and tumor size (f) in 90 patients with HCC (log-rank test). Probability of survival of patients: (c) low expression of AGO2 (*n* = 26) vs. overexpression of AGO2 (*n* = 61) (*P* = 0.037); (d) age > 65 (*n* = 12) vs. age≦65 (*n* = 77) (*P* = 0.597); (e) male (*n* = 81) vs. female (*n* = 9) (*P* = 0.625); (f) tumor size > 5 cm (*n* = 50) vs. tumor size≦5 cm (*n* = 39) (*P* = 0.005).

To explore AGO2 expression in HCC, we first checked the AGO2 expression in the HCC tissue microarray. Immunohistochemical staining was used to detect AGO2 protein in matched pairs of HCC and noncancerous tissues. The representative images (Figure 1(a)) showed high expression of AGO2 in HCC tissue and low expression in normal tissue. The AGO2 expression levels were scored according to the staining intensity ranging from 0 to 3 (0, 1, 2, and 3). Several missing or damaged samples were excluded during immunohistochemistry tissue preparation, and a final total of 85 cases were included in the statistical analysis. Result showed that in 80 of 85 (94%) matched tissue sets, AGO2 expression was significantly higher in tumor tissues than in normal tissues (Figure 1(b), Table 1). Table 1 shows the correlation of AGO2 expression with clinical parameters of HCC patients. Data demonstrated that AGO2 expression in the human HCC tissues was only associated with tumor size (*P* = 0.011) of the patients. Subsequently, the correlation between AGO2 expression and outcome of patients was also assessed. Kaplan-Meier analysis revealed that high level of AGO2 was related to significantly poor overall survival (OS) (Figure 1(c)). Other clinicopathological characteristics, such as tumor size, could also affect the OS, while age and gender showed no significant correlation with OS (Figures 1(d)–1(f)). Altogether, these data support that AGO2 associated with poor prognosis in HCC patients.

### 3.2. Knockout of AGO2 Inhibited Cell Proliferation and Migration in HCC In Vitro

**Figure 2:**
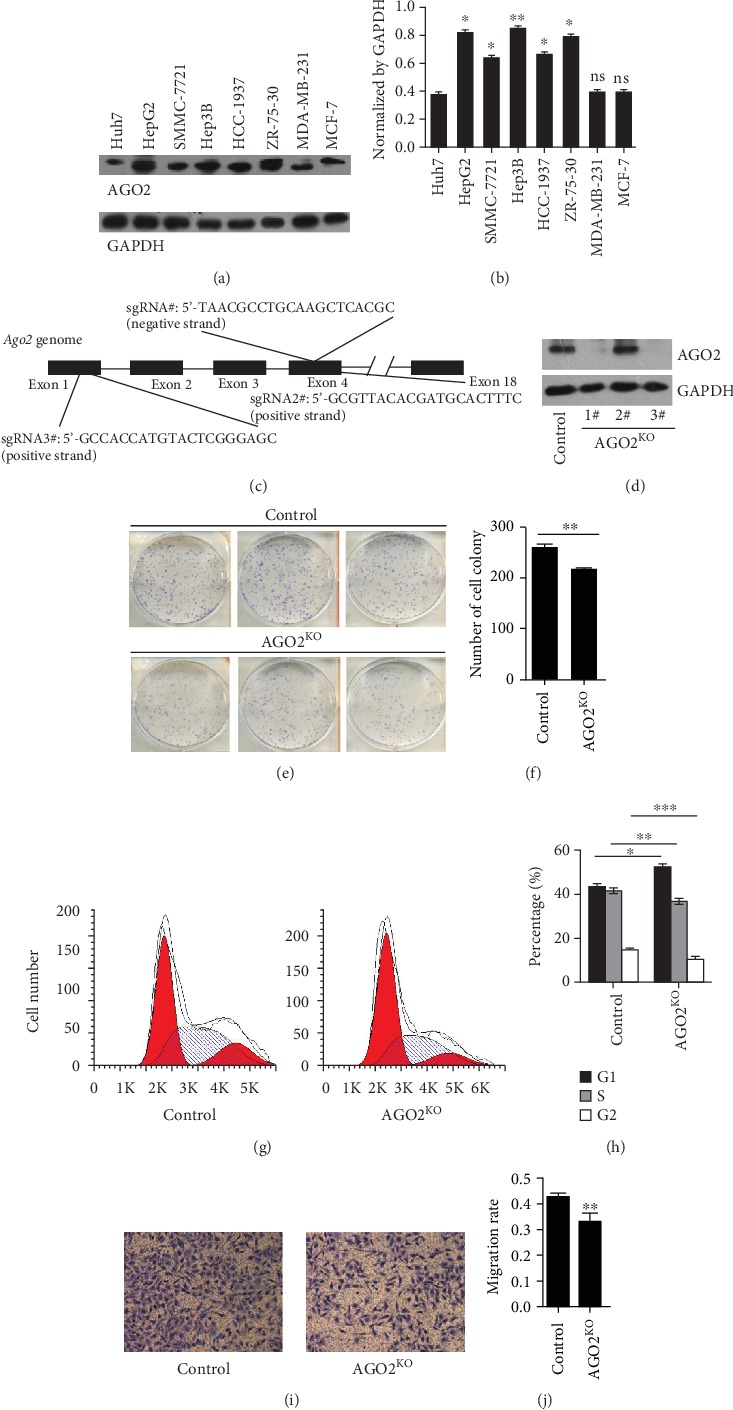
AGO2 promoted cell proliferation and migration in HCC in vitro. (a) AGO2 protein abundances in different cell lines were detected by western blot analysis; GAPDH was used as a loading control. (b) The ratio of AGO2 to GAPDH in each cell line was measured by densitometric analysis of western blot bands, respectively. (c) Designation of AGO2 protein depletion in SMMC-7721 cells using CRISPR/Cas9 gene editing strategy. (d) AGO2 expression in SMMC-7721 cells was determined by western blotting. (e) Colony formation detected by Giemsa staining for normal and AGO2^−/−^ SMMC-7721 cells. (f) AGO2 knockout induced inhibition in colony formation as compared to control. (g) Diagram of the cell cycle analysis for normal and AGO2^−/−^ SMMC-7721 cells based on flow cytometry. (h) The proportions of cells in G1, S, and G2 phases were calculated. (i) Representative images for cell migration assay. (j) Numbers of stained cells in migration assay were counted. Data are normalized and expressed as the fold change relative to control values. Values represented as the means ± S.D. (*n* > 3 each group); ^∗^*P* < 0.05, ^∗∗^*P* < 0.01, and ^∗∗∗^*P* < 0.005.

To explore the biological function of AGO2 in HCC, we evaluated the AGO2 expression in several cell lines (Figures 2(a) and 2(b)). Interestingly, western blot analysis and qRT-PCR examination revealed different expression patterns of AGO2 protein and mRNA in several cell lines. For example, Huh-7 had the lowest AGO2 protein abundance (Figures 2(a) and 2(b)) and the highest mRNA expression (Supplementary Figure 1A and 1B). In addition, compared with other cell lines, HepG2 and ZR-75-30 cells showed high AGO2 protein expression but conversely low mRNA levels (*P* < 0.05, Figure 2(a) and Supplementary Figure 1B). This result suggested AGO2 protein abundance was not correlated well with mRNA expression in these cell lines. In comparison, relatively high correlation between AGO2 protein and mRNA levels was observed in two liver cancer cell lines. SMMC-7221 and Hep3B had moderately high expression of AGO2 at both protein and mRNA levels.

Subsequently, using CRISPR/Cas9 genome editing technology (Figure 2(c)), we successfully screened out several AGO2 knockout (AGR2^−/−^) clones of SMMC-7721 cells. The effect of knockout of AGO2 was confirmed by western blot analysis (Figure 2(d)). Colony formation assay revealed that knockout of *AGO2* significantly repressed cell proliferation (Figures 2(e) and 2(f) , *P* < 0.01). Cell cycle analysis showed that SMMC-7721 *AGO2*^−/−^ cells had increased percentage of cell numbers in the G0/G1 (G1) phase but reduced distribution in S and G2/M (G2) phases (Figures 2(g) and 2(h), *P* < 0.01), suggesting a cell cycle shift from S and G2/M to G0/G1 phases. Knockout of *AGO2* also significantly inhibited cell migration, as indicated by the transwell assay (Figures 2(i) and 2(j), *P* < 0.01). In addition, *AGO2* knockdown was performed by transfecting siRNA into Hep3B cells (Supplementary Figure 2A), as screening of *AGO2*^−/−^ Hep3B clones failed by using CRISPR/Cas9-mediated gene knockout. The result showed that *AGO2* knockdown obviously inhibited cell proliferation in Hep3B when compared with control Hep3B cells (Supplementary Figure 2B, *P* < 0.05). Taken together, these results demonstrated that knockout of AGO2 inhibits cell tumorigenesis in HCC *in vitro*.

### 3.3. Knockout of AGO2 Suppressed HCC Tumor Growth and Size In Vivo

**Figure 3:**
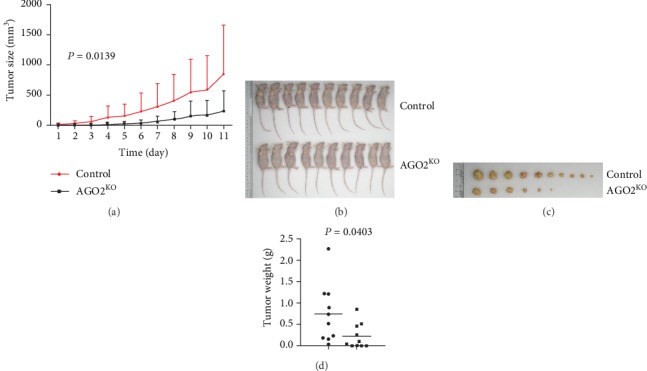
AGO2 enhanced tumor growth and size in HCC in vivo. (a) Control and AGO2 knockout SMMC-7721 cells were subcutaneously injected in the flank of BALB/c nude mice with 2 × 10^6^ cells. About 7 days post injection, the tumor size was measured every other day. The tumor size was calculated as (length × width^2^)/2. At the indicated time, all mice were sacrificed and the tumors were photographed. (b) The size of killed nude mice of the indicated groups. (c) The size of tumors isolated from nude mice. (d) The weights of tumors were measured. Values represented as the means ± S.D.

To further investigate the AGO2 role in regulating xenograft tumor growth, nude mice were injected subcutaneously with SMMC-7721 and *AGO2*^−/−^ cells, respectively. The body weights of mice bearing the tumors were not significantly changed (not shown) within 2 weeks. Tested nude mice (*n* = 10/group) developed subcutaneous tumors with a size of approximately 0.90 to 840 mm^3^ after injection with SMMC-7721 or SMMC-7721 *AGO2*^−/−^ cells (2 × 10^6^/mouse). SMMC-7721 control cells generated visible tumors at day 3 and formed a continuously growing mass. However, SMMC-7721 *AGO2*^−/−^ cells generated visible tumors at day 6, and the tumor growth rate of SMMC-7721 *AGO2*^−/−^ cells was lower compared with control cells (Figure 3(a)). These findings suggested that AGO2 promoted tumor growth *in vivo*, while the knockout of AGO2 appeared to not affect the mice growth (Figure 3(b)). The tumor sizes of mice bearing with SMMC-7721 control cells were obviously smaller than those with SMMC-7721 *AGO2*^−/−^ cells (Figure 3(c)). Accordingly, the average tumor weight in the SMMC-7721 control group was heavier than that in the SMMC-7721 *AGO2*^−/−^ group (Figure 3(d)). Therefore, knockout of *AGO2* suppressed tumor growth and size in HCC *in vivo.*

### 3.4. AGO2 Regulated Expression of Survivin, Vimentin, and Snail

**Figure 4:**
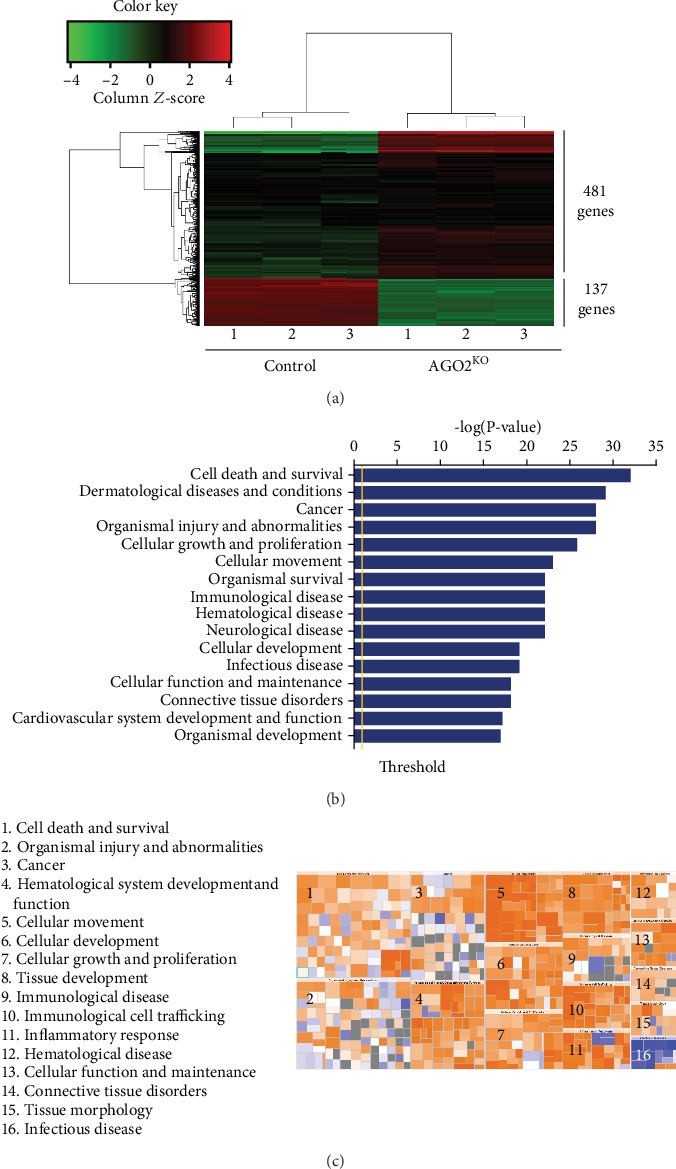
Bioinformatics analysis of microarray data. (a) The heat map of the pairwise comparison in control and AGO2 knockout SMMC-7721 cells. The colored bar shows the expression levels of genes. (b) Gene Ontology (GO) functional classification of differentially expressed genes (DEGs). The GO terms are shown in the bar chart of biological processes, cellular components, and molecular functions. (c) Heatmaps showed the upregulation and downregulation of DEGs associated with disease. Orange: *Z*‐score > 0, blue: *Z*‐score < 0, gray: no *Z*-score value. *Z*‐score > 2 indicates that the function is significantly activated, while *Z*‐score < ‐2 indicates that the function is significantly inhibited.

To explore the downstream pathways of AGO2 involved in HCC tumorigenesis, we performed microarray profiling of gene expression. We initially evaluated the global transcriptomic changes associated with depletion of *AGO2* in SMMC-7721 cells. Heatmaps for gene expression exhibited a list of 2,327 genes showing >2-fold differential expression (Figure 4(a)). Gene ontology (GO) enrichment analysis revealed that these differentially expressed genes (DEG) were mainly enriched in the pathways that control cell behaviors, including cell death and survival, cellular growth, and proliferation, as well as cellular movement (Figures 4(b) and 4(c)). Further comparative analysis of DEGs between *AGO2*^−/−^ and control SMMC-7721 cells showed remarkably decreased expression of two clusters of genes involved in cell proliferation (e.g., *Survivin*) and cell metastasis (e.g., *Snail* and *Vimentin*).

**Figure 5:**
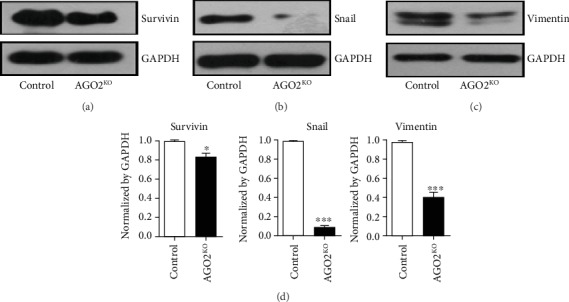
AGO2 upregulated Survivin, Vimentin, and Snail expression. Western blot results showed that the expression of Survivin (a), Vimentin (b), and Snail (c) were significantly decreased in SMMC-7721 *AGO2*^−/−^ cells compared with control cells. GAPDH was used as a loading control. (d) Quantification of protein expression of Survivin (a), Vimentin (b), and Snail (c) that was normalized by GAPDH, respectively. Values represented as the means ± S.D. (*n* > 3 each group); ^∗^*P* < 0.05, ^∗∗^*P* < 0.01, and ^∗∗∗^*P* < 0.005.

It has been reported that Survivin, Vimentin, and Snail play an important role in cell proliferation and metastasis of hepatocellular carcinoma [16, 17]. A western blot test confirmed that Survivin, Vimentin, and Snail expression dramatically declined in SMMC-7721 *AGO2*^−/−^ cells when compared with control cells (Figures 5(a)–5(d)). Especially, Vimentin was decreased by around 60% and Snail was decreased by 90% due to knockout of AGO2 (Figure 5(d)). Moreover, expression of Survivin, Vimentin, and Snail proteins were significantly reduced in Hep3B cells transfected with *AGO2* siRNA (Supplementary Figure 3A-C, *P* < 0.05). These data indicated that AGO2 might promote hepatocellular proliferation and migration through regulating Survivin, Vimentin, and Snail in the development of hepatocellular carcinoma.

### 3.5. Overexpression of AGO2 Enhanced HCC Growth and Migration In Vitro

**Figure 6:**
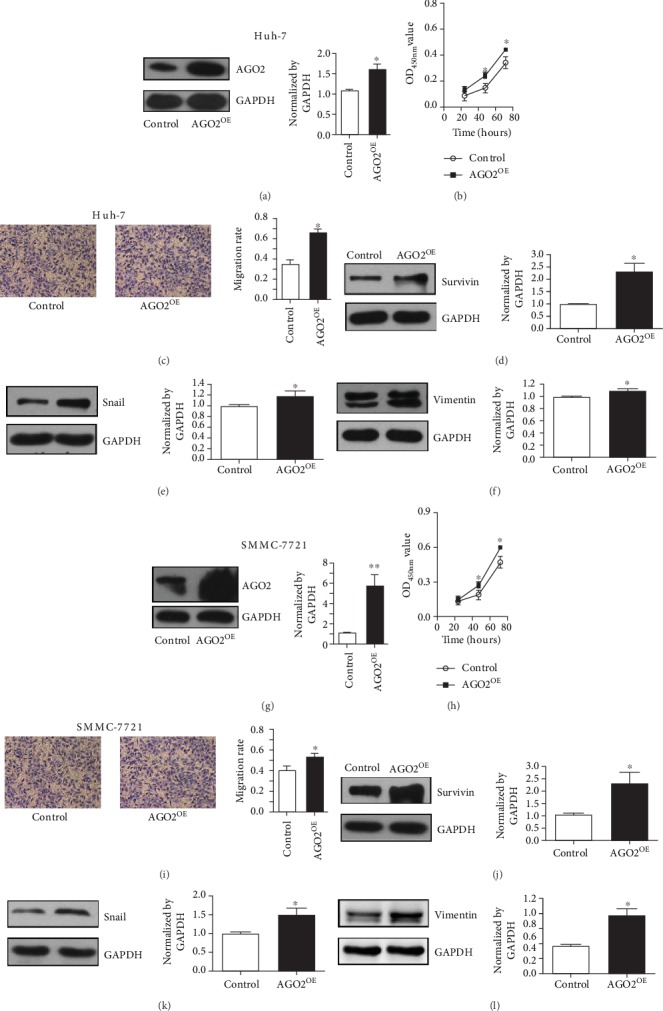
AGO2 overexpression promoted proliferation and migration of SMMC-7721 and Huh-7 in vitro. (a–f) Huh-7 cells were transiently transfected with pCMV3-AGO2 (AGO2^OE^) and an empty vector pCMV3 (control), respectively. (g–l) SMMC-7721 cells were used for AGO2 overexpression and subsequently analysis. Western blot was used to examine the effect of pCMV3-AGO2 transfection into Huh-7 (a) and SMMC-7721 (g) cells. (b and h) CCK8 assay was performed to detect cell proliferation of AGO2^OE^ Huh-7 (b) and SMMC-7721 (h) cells. (c and i) Cell migration rates of AGO2^OE^ Huh-7 (c) and SMMC-7721 (i) were determined by transwell assay. Western blot was performed to detect the effect of AGO2 overexpression on Survivin, Snail, and Vimentin expression in Huh-7 (d–f) and SMMC-7721 (j–l) cells. The ratio of Survivin, Snail, and Vimentin to GAPDH in Huh-7 and SMMC-7721 was measured by densitometric analysis of western blot bands, respectively. Values represented as the means ± S.D. (*n* > 3 each group); ^∗^*P* < 0.05, ^∗∗^*P* < 0.01, and ^∗∗∗^*P* < 0.005.

SMMC-7221 and another HCC cell line Huh-7, which showed relatively low AGO2 protein expression, were transiently transfected with pCMV3-AGO2 and control pCMV3 vector, respectively. Increased production of AGO2 in both SMMC-7221 and Huh-7 was detected by western blot analysis 48 hours post transfection (Figures 6(a) and 6(g)). To evaluate the effect of overexpression, the CCK8 cell proliferation test and transwell assay were performed. The results showed that AGO2 overexpression (AGO^OE^) significantly promoted cell proliferation and migration of Huh-7 (Figures 6(a)–6(c), *P* < 0.05) and SMMC-7221 (Figures 6(g)–6(i), *P* < 0.05). Furthermore, the expression of Survivin, Vimentin, and Snail was also determined. Result showed that Survivin, Vimentin, and Snail were significantly enhanced in AGO^OE^ Huh-7 (Figures 6(d)–6(f), *P* < 0.05) and SMMC-7221 (Figures 6(j)–6(l), *P* < 0.05) compared with cells transfected with the control vector. These results supported the speculation that AGO2 facilitated cell proliferation and migration by upregulating Survivin and Vimentin/Snail in HCC.

## 4. Discussion

*AGO2* was first identified as an oncogene in renal cell carcinoma. It has been reported that the single-nucleotide polymorphism (SNP) of *AGO2* was related to the tumorigenesis of renal cell carcinoma [18]. Abnormal overexpression of AGO2 has been found in several human tumors, including breast cancer [19], urothelial carcinoma of the bladder [11], glioma [12], and nasopharyngeal carcinoma [10]. Accumulation of AGO2 is generally associated with higher tumor grading and poorer prognosis [3, 20]. The present study showed that AGO2 expressed at high levels in the tumors isolated from HCC patients, which is consistent with previous studies [13]. Our study also showed that AGO2 was associated with lower overall survival of the HCC patients, suggesting that AGO2 could serve as an independent predictor for worse clinical outcomes.

It has been well established that AGO2 is important for the developmental processes, including neural tube closure, cardiac failure, and embryonic lethality [6, 21]. Recently, increasing evidences have demonstrated that AGO2 also plays multifunctional roles in tumorigenesis and progression. In this study, we generated *AGO2* knockout SMMC-7721 cells by using CRISPR/Cas9-based gene editing technology. The depletion of AGO2 obviously reduced cell proliferation and migration, as previously reported [9, 13]. Parallel results were observed from *in vivo* experiments. Mice bearing *AGO2^−/−^* xenografts showed decreased tumor sizes and delayed tumor progression. These data confirmed that AGO2 could be a promising indicator for prognostic prediction of HCC.

With nuclease and ribonuclease activity [22, 23], AGO2 generally affects tumorigenesis by regulating miRNA processing and maturation. Despite AGO2 being an essential mediator of miRNA function, recent studies have also revealed that AGO2 could directly interact with mRNA of famous oncogenes via miRNA-independent ways [20]. For instance, it has been proved that AGO2 could bind to the promoter of *FAK* (focal adhesion kinase), leading to the improvement of HCC metastasis [13]. Aberrant expression of multiple genes might contribute to HCC development and progression [24, 25]. Therefore, we further determined the AGO2-associated gene expression profiling by performing mRNAs microarray analysis using native and gene-modified *AGO2*^−/−^ SMMC-7721 cells. The bioinformatics analysis showed a potential role of AGO2 in regulating a series of mRNA transcription, especially of which related to the cell proliferation and migration. Knockout of *AGO2* conducted by CRISPR/Cas-9 system significantly reduced the expression of Survivin, Vimentin, and Snail in both mRNA and protein levels. Survivin was found crucial in mediating cell proliferation and survival of hepatocellular carcinoma [16, 26]. Vimentin and Snail are typical mesenchymal biomarker proteins that could promote HCC metastasis [27]. Our results suggested the participation of these molecules in AGO2 promoting HCC progression. A future work could further illustrate the molecular mechanisms how AGO2 regulates the expression of Survivin, Vimentin, and Snail.

## 5. Conclusions

In conclusion, high expression of AGO2 is a potential indicator for prognostic prediction of HCC. Disruption of *AGO2* gene expression led to inhibition of HCC tumor proliferation and metastasis *in vitro* and *in vivo*. The depletion of *AGO2* could remarkably decrease the expression of two clusters of genes involved in cell proliferation (e.g., *Survivin*) and cell metastasis (e.g., *Snail*, *Vimentin*), respectively. This study not only explored the role of AGO2 in the tumorigenesis and progression in HCC but also revealed that AGO2 might facilitate HCC tumorigenesis through modulating expression of Survivin, Vimentin, and Snail.

## Figures and Tables

**Table 1:** The correlation of AGO2 expression with clinical parameters of HCC patients. CC, correlation coefficient.

Clinical data	Samples (90)	AGO2 expression in HCC tissues					AGO2 expression in noncancerous tissue				
		Low (26)	High (61)	Lost (3)	CC	*P* value	Low (85)	High (3)	Lost (2)	CC	*P* value
Ages					-0.001	0.989				0.017	0.876
≤65	77	22	53	2			72	3	2		
>65	12	4	7	1			12	0	0		
Lost	1	0	1	0			1	0	0		
Tumor size					-0.272	0.011				0.045	0.677
≤5 cm	39	8	30	1			38	1	0		
>5 cm	50	18	30	2			46	2	2		
Lost	1	0	1	0			1	0	0		
Gender					0.081	0.454				0.014	0.895
Male	81	24	54	3			76	3	2		
Female	9	2	7	0			9	0	0		
Lost	0	0	0	0			0	0	0		
Grade					0.064	0.559				0.106	0.327
I	3	1	2	0			3	0	0		
II	54	17	35	2			51	1	2		
III	33	8	24	1			31	2	0		
Lost	0	0	0	0			0	0	0		
T staging					-0.147	0.195				0.205	0.068
T1	11	3	7	1			11	0	0		
T2	29	6	23	0			28	0	1		
T3	39	14	23	2			36	2	1		
T4	3	0	3	0			3	0	0		
Lost	8	3	5	0			7	1	0		
N staging					-0.043	0.703				0.006	0.959
N0	81	23	55	3			77	2	2		
NI	1	0	1	0			1	0	0		
Lost	8	3	5	0			7	1	0		
M staging					-0.139	0.215				0.006	0.959
M0	82	23	56	3			78	2	2		
M1	1	1	0	0			1	0	0		
Lost	7	2	5	0			6	1	0		

## Data Availability

The data used to support the findings of this study are available from the corresponding author upon request.

## Abbreviations

AGO2: Argonaute RISC Catalytic Component 2
HCC: Hepatocellular carcinoma
CRISPR: Clustered regularly interspaced short palindromic repeats
eIF2C: Eukaryotic translation initiation factor 2C
GAPDH: Glyceraldehyde-3-Phosphate Dehydrogenase
DMEM: Dulbecco's Modified Eagle Medium
CCK8: Cell Counting Kit-8
BCA kit: Bicinchoninic acid assay kit
TBST: Tris-Buffered Saline with Tween 20.

## Supplementary Materials

Supplementary Materials Supplementary Figure 1. Detection of AGO2 mRNA expression in different cell lines. (A) Expression levels of AGO2 in liver cancer cell lines and breast cancer cell lines were measured using quantitative PCR, respectively. (B) Relative AGO2 mRNA expression was quantified and normalized by housekeeping gene GAPDH. Data are expressed as fold change of AGO2 expression in individual cell lines with respect to that in MCF-7. Values represented as means ± S.D., n > 3 each group, ∗*P* < 0.05, ∗∗*P* < 0.01, ∗∗∗*P* < 0.005. Supplementary Figure 2. Knock-down of AGO2 inhibits cell proliferation in Hep3B cell line. (A-B) Knock-down of AGO2 expression was performed by transiently transfection of siRNA into Hep3B. The analysis of AGO2 mRNA (A) and protein (B) expression was determined by qRT-PCR and western blotting after 48 h of transfection, respectively. Control-siRNA (nonspecific), 5′-TTCTCCGAACGTGTCACGT-3′; AGO2-siRNA1, 5′-GCACAGCCAGTAATCGAGTTT-3′; AGO2-siRNA2, 5′-CAATCAAATTACAGGCCAATT-3′. (C) Colony formation was detected by giemsa staining for Hep3B cells transfected with control-siRNA and AGO2-siRNA1, respectively. AGO2 knock-down induced inhibition in colony formation as compared to control. Data are normalized and expressed as fold change relative to control values. Values represented as means ± S.D., *n* > 3 each group, ∗*P* < 0.05, ∗∗*P* < 0.01, ∗∗∗*P* < 0.005. Supplementary Figure 3. Knock-down of AGO2 decreases expression of Survivin, Vimentin and Snail in Hep3B. Western blot analysis showed that the expression of Survivin (A), Vimentin (B) and Snail (C) were significantly decreased in Hep3B cells transfected with AGO2-siRNA1 compared to control cells. At 48 h post transfection, the tests were performed in three independent cell culture preparations. GAPDH was used as a loading control. Quantification of protein expression of Survivin (A), Vimentin (B) and Snail (C) that was normalized by GAPDH respectively. Values represented as means ± S.D., ∗*P* < 0.05, ∗∗*P* < 0.01, ∗∗∗*P* < 0.005.
