# Supplementary material for: Accumulation of AGO2 Facilitates Tumorigenesis of Human Hepatocellular Carcinoma
Source: Biomed Res Int. 2020 Apr 30;2020:1631843. doi: 10.1155/2020/1631843 (PMC7210519; doi:10.1155/2020/1631843)
Supplement: Supplementary Materials — Supplementary Figure 1. Detection of AGO2 mRNA expression in different cell lines. (A) Expression levels of AGO2 in liver cancer cell lines and breast cancer cell lines were measured using quantitative PCR, respectively. (B) Relative AGO2 mRNA expression was quantified and normalized by housekeeping gene GAPDH. Data are expressed as fold change of AGO2 expression in individual cell lines with respect to that in MCF-7. Values represented as means ± S.D., n > 3 each group, ∗P < 0.05, ∗∗P < 0.01, ∗∗∗P < 0.005. Supplementary Figure 2. Knock-down of AGO2 inhibits cell proliferation in Hep3B cell line. (A-B) Knock-down of AGO2 expression was performed by transiently transfection of siRNA into Hep3B. The analysis of AGO2 mRNA (A) and protein (B) expression was determined by qRT-PCR and western blotting after 48 h of transfection, respectively. Control-siRNA (nonspecific), 5′-TTCTCCGAACGTGTCACGT-3′; AGO2-siRNA1, 5′-GCACAGCCAGTAATCGAGTTT-3′; AGO2-siRNA2, 5′-CAATCAAATTACAGGCCAATT-3′. (C) Colony formation was detected by giemsa staining for Hep3B cells transfected with control-siRNA and AGO2-siRNA1, respectively. AGO2 knock-down induced inhibition in colony formation as compared to control. Data are normalized and expressed as fold change relative to control values. Values represented as means ± S.D., n > 3 each group, ∗P < 0.05, ∗∗P < 0.01, ∗∗∗P < 0.005. Supplementary Figure 3. Knock-down of AGO2 decreases expression of Survivin, Vimentin and Snail in Hep3B. Western blot analysis showed that the expression of Survivin (A), Vimentin (B) and Snail (C) were significantly decreased in Hep3B cells transfected with AGO2-siRNA1 compared to control cells. At 48 h post transfection, the tests were performed in three independent cell culture preparations. GAPDH was used as a loading control. Quantification of protein expression of Survivin (A), Vimentin (B) and Snail (C) that was normalized by GAPDH respectively. Values represented as means ± S.D., ∗P < 0.05, ∗∗P < 0.01, ∗∗∗P < 0.005. [file 1631843.f1.pptx]

## Slide 1
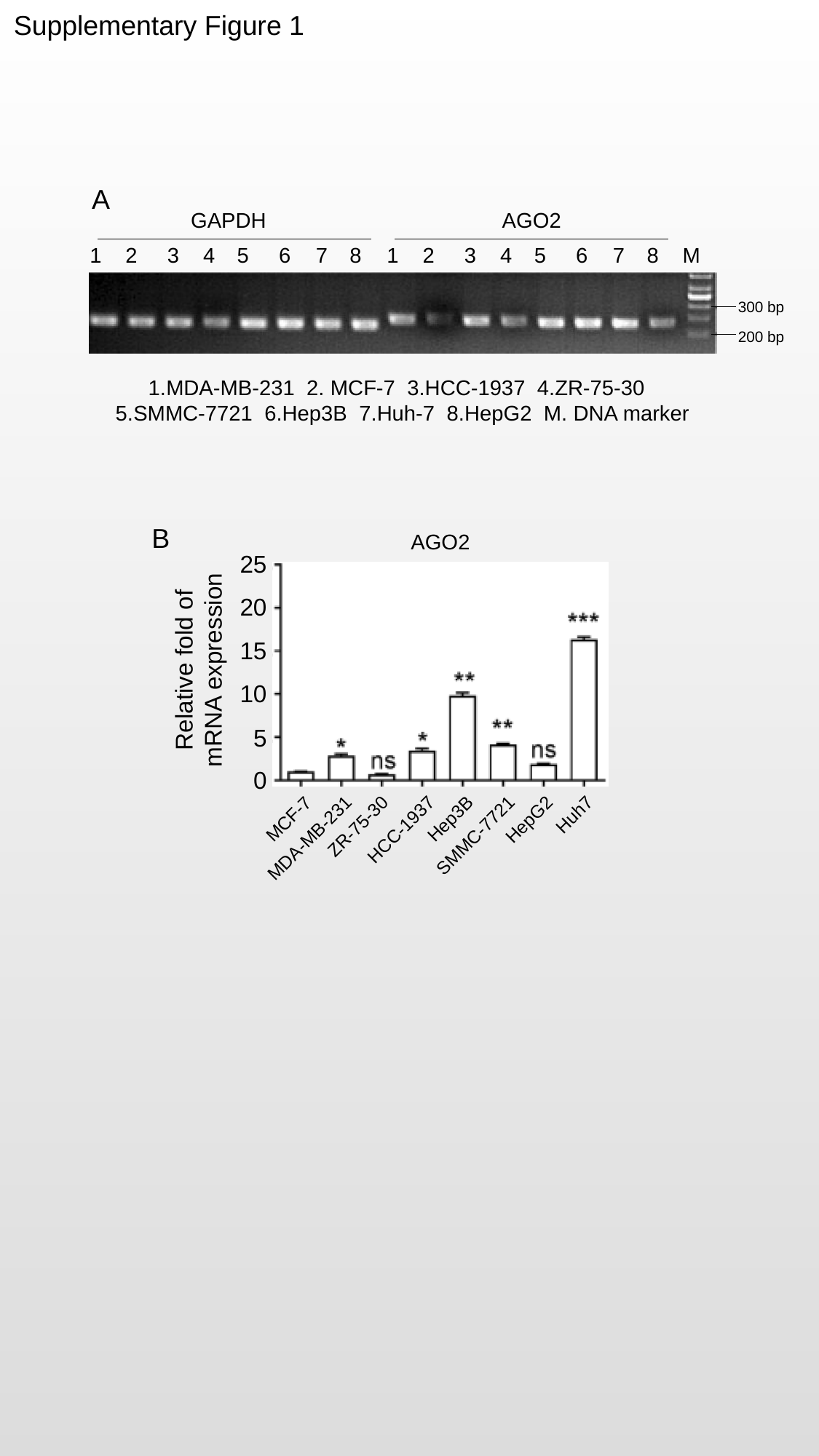

Supplementary Figure 1
A
GAPDH
AGO2
1 2 3 4 5 6 7 8
1 2 3 4 5 6 7 8 M
300 bp
200 bp
1.MDA-MB-231 2. MCF-7 3.HCC-1937 4.ZR-75-30
5.SMMC-7721 6.Hep3B 7.Huh-7 8.HepG2 M. DNA marker
B
AGO2
25
20
15
10
5
0
Relative fold of
mRNA expression
Huh7
Hep3B
MCF-7
HepG2
ZR-75-30
HCC-1937
SMMC-7721
MDA-MB-231

## Slide 2
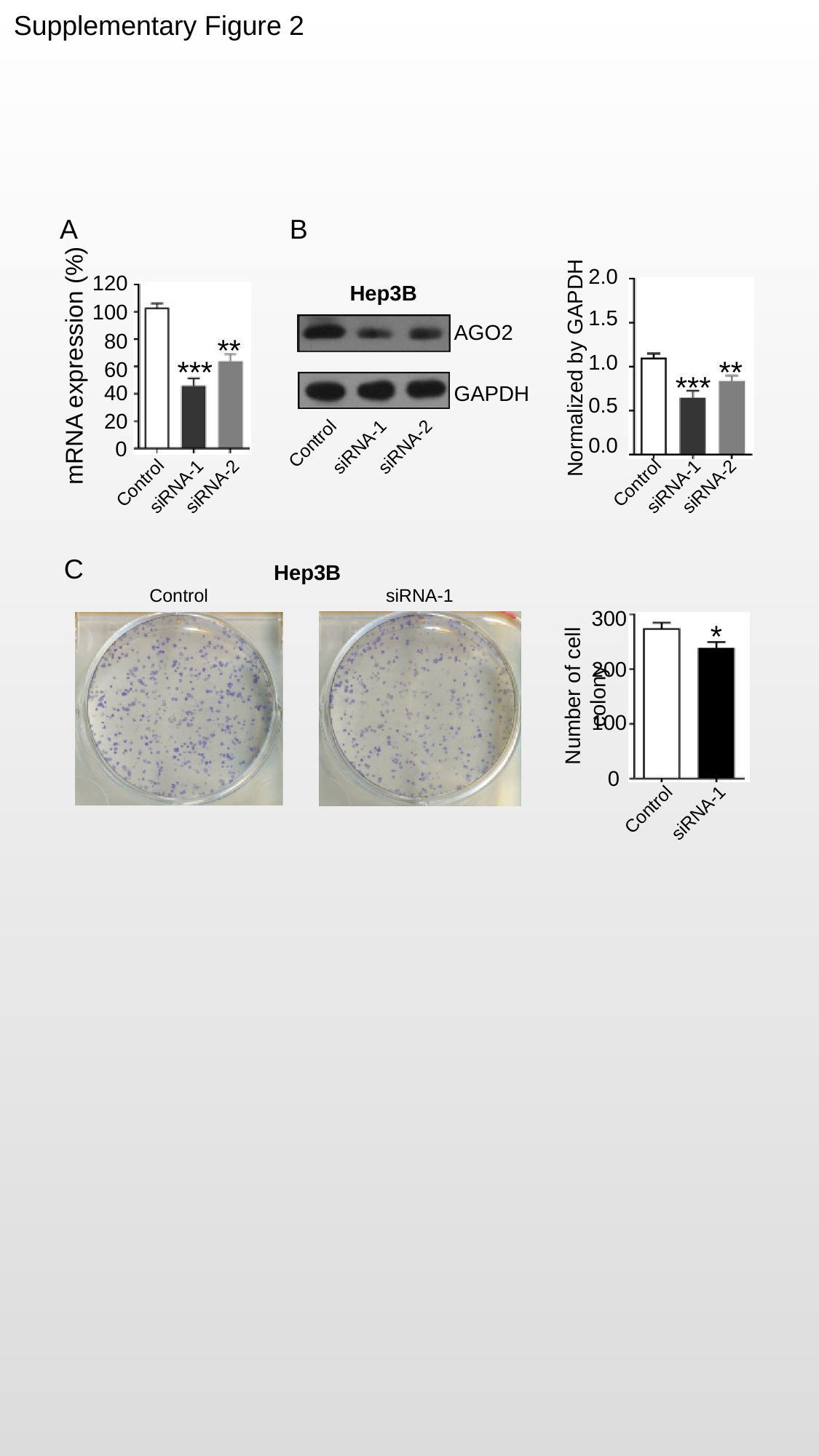

Supplementary Figure 2
A
B
2.0
1.5
1.0
0.5
0.0
120
Hep3B
100
AGO2
80
**
mRNA expression (%)
***
**
Normalized by GAPDH
60
***
40
GAPDH
20
Control
siRNA-1
siRNA-2
0
Control
siRNA-1
siRNA-2
Control
siRNA-1
siRNA-2
C
Hep3B
Control
siRNA-1
300
200
100
0
*
Number of cell colony
Control
siRNA-1

## Slide 3
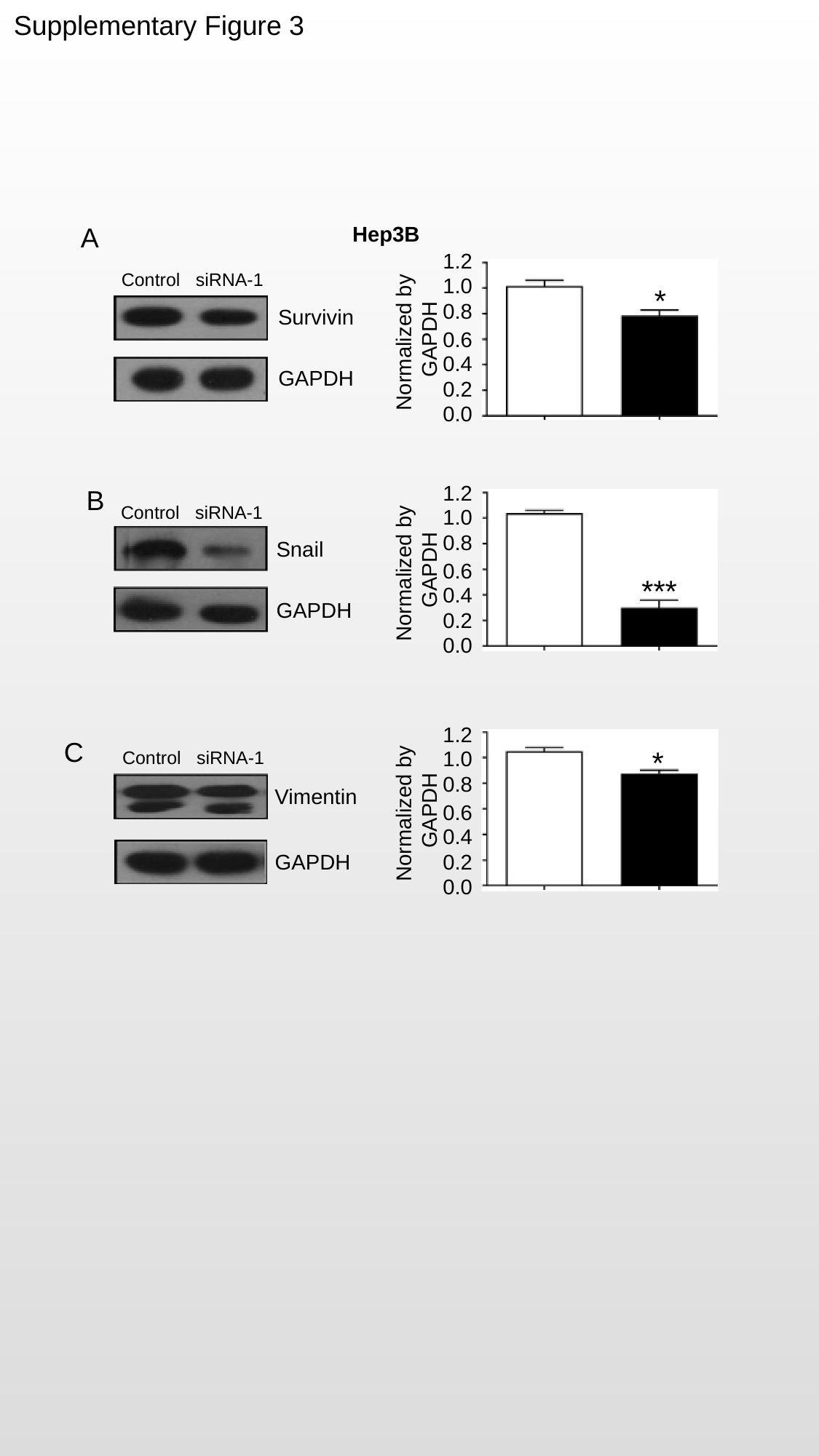

Supplementary Figure 3
A
Hep3B
1.2
1.0
0.8
0.6
0.4
0.2
0.0
Control
siRNA-1
*
Survivin
Normalized by
GAPDH
GAPDH
1.2
1.0
0.8
0.6
0.4
0.2
0.0
B
Control
siRNA-1
Snail
Normalized by
GAPDH
***
GAPDH
1.2
1.0
0.8
0.6
0.4
0.2
0.0
C
*
Control
siRNA-1
Vimentin
Normalized by
GAPDH
GAPDH
